# Metabolic engineering of *Rhodococcus jostii* RHA1 for production of pyridine-dicarboxylic acids from lignin

**DOI:** 10.1186/s12934-020-01504-z

**Published:** 2021-01-19

**Authors:** Edward M. Spence, Leonides Calvo-Bado, Paul Mines, Timothy D. H. Bugg

**Affiliations:** 1 Department of Chemistry, University of Warwick, Coventry, CV4 7AL UK; 2 Biome Bioplastics Ltd, North Road, Marchwood, Southampton, SO40 4BL UK

**Keywords:** Lignin degradation, Pyridine dicarboxylic acid, Metabolic engineering, *Rhodococcus jostii* RHA1, Gene promoter

## Abstract

Genetic modification of *Rhodococcus jostii* RHA1 was carried out in order to optimise the production of pyridine-2,4-dicarboxylic acid and pyridine-2,5-dicarboxylic acid bioproducts from lignin or lignocellulose breakdown, via insertion of either the *Sphingobium* SYK-6 *ligAB* genes or *Paenibacillus praA* gene respectively. Insertion of inducible plasmid pTipQC2 expression vector containing either *ligAB* or *praA* genes into a Δ*pcaHG R. jostii* RHA1 gene deletion strain gave 2–threefold higher titres of PDCA production from lignocellulose (200–287 mg/L), compared to plasmid expression in wild-type *R. jostii* RHA1. The *ligAB* genes were inserted in place of the chromosomal *pcaHG* genes encoding protocatechuate 3,4-dioxygenase, under the control of inducible P_icl_ or P_nitA_ promoters, or a constitutive P_tpc5_ promoter, producing 2,4-PDCA products using either wheat straw lignocellulose or commercial soda lignin as carbon source. Insertion of *Amycolatopsis* sp. 75iv2 *dyp2* gene on a pTipQC2 expression plasmid led to enhanced titres of 2,4-PDCA products, due to enhanced rate of lignin degradation. Growth in minimal media containing wheat straw lignocellulose led to the production of 2,4-PDCA in 330 mg/L titre in 40 h, with > tenfold enhanced productivity, compared with plasmid-based expression of *ligAB* genes in wild-type *R. jostii* RHA1. Production of 2,4-PDCA was also observed using several different polymeric lignins as carbon sources, and a titre of 240 mg/L was observed using a commercially available soda lignin as feedstock.
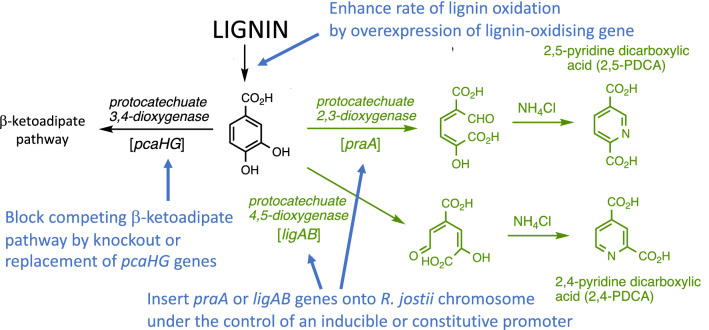

## Background

The aromatic heteropolymer lignin accounts for 15–25% of plant cell wall lignocellulose, and is the most abundant renewable source of aromatic carbon in the biosphere. The conversion of lignocellulose from plant biomass into fuels and chemicals via the biorefinery concept requires the efficient conversion of cellulose, hemi-cellulose and lignin into high value products, but at present the majority of lignin produced by pulp/paper manufacture and biofuel production is burnt for energy. Hence there is considerable interest in the biocatalytic or chemocatalytic valorisation of lignin into useful chemical products [1].

One strategy for biocatalytic valorisation of lignin that has shown promise is to engineer lignin-degrading micro-organisms to produce target chemicals [2]. A gene deletion mutant of *Rhodococcus jostii* RHA1 in which the vanillin dehydrogenase gene was deleted was shown in 2013 to generate vanillin as a bio-product when grown on minimal media containing wheat straw lignocellulose [3]. Metabolic funnelling of monocyclic lignin breakdown products via protocatechuic acid and subsequent metabolism via the β-ketoadipate pathway has facilitated the metabolic engineering of *Pseudomonas putida* KT2440 to produce polyhydroxyalkanoic acids [4, 5] and *cis,cis*-muconic acid, which can be chemically converted to adipic acid [6]. Metabolic engineering of *Corynebacterium glutamicum* to produce *cis,cis*-muconic acid has also been reported [7].

**Fig. 1:**
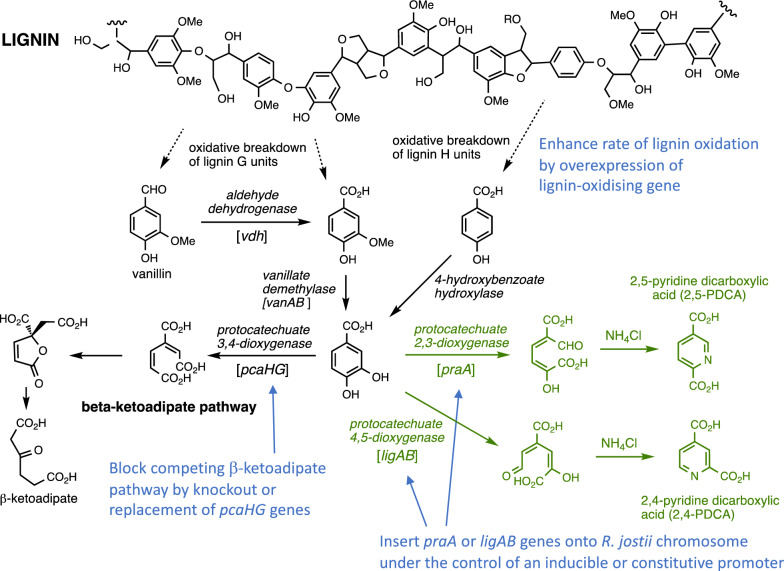
Generation of pyridine-dicarboxylic acid bioproducts (in green) via re-routing of aromatic degradation pathways in *R. jostii* RHA1, and the aims of the metabolic engineering study (in blue). Gene designations shown in brackets

We have previously reported that re-routing of protocatechuic acid via either protocatechuate 4,5-dioxygenase (*Sphingobium* SYK-6 *ligAB* genes) or protocatechuate 2,3-dioxygenase (*Paenibacillus praA* gene) in *R. jostii* RHA1, followed by ammonia cyclisation of the extradiol ring fission product, generates pyridine-2,4-dicarboxylic acid (2,4-PDCA) or pyridine-2,5-dicarboxylic acid (2,5-PDCA) bioproducts respectively (see Fig. 1), in titres of 90–125 mg/L cell culture, in M9 minimal media containing either 0.1% vanillic acid or 1% wheat straw lignocellulose [8]. These pyridine-dicarboxylic acid products are analogues of terephthalic acid that could potentially be converted into new polyester bioplastics [8], hence we wished to engineer stable high-yielding strains of *R. jostii* RHA1 that could be used to generate PDCA products via bioconversion of lignin-containing feedstocks. In the previous study, the *ligAB* or *praA* genes were introduced on an inducible pTipQC2 plasmid [8], using thiostrepton as inducer [9]. The aims of this metabolic engineering study (see Fig. 1) were: (1) to integrate the *ligAB* or *praA* genes onto the chromosome of *R. jostii* RHA1 under the control of an inducible or constitutive promoter; (2) to block the competing β-ketoadipate pathway which metabolises protocatechuic acid; (3) to enhance the metabolic flux of lignin depolymerisation via overexpression of lignin-oxidising genes.

We have also investigated the use of a commercially available soda lignin, Green Value Protobind P1000, as a feedstock for microbial bioconversion. We have previously used milled wheat straw lignocellulose or commercially available Kraft lignin as feedstocks [8]. Other research groups have used alkali-pretreated lignin (APL) as feedstock, which contains a high proportion of p-hydroxycinnamic acid monomers [4–6]. There are several different types of lignin preparation, which vary in their exact molecular structure, molecular weight, and solubility, and comparative studies have shown that lignins rich in b-O-4 lignin units, such as organosolv lignin, generally give higher conversion yields for chemocatalytic and biocatalytic transformation [10], although the low solubility of organosolv lignin hinders its application for biotransformation, and it is not commercially available.

## Results

### Gene deletion of pcaHG genes encoding protocatechuate 3,4-dioxygenase

We first investigated deletion of the *pcaHG* genes encoding protocatechuate 3,4-dioxygenase, the first enzyme of the β-ketoadipate pathway for metabolism of protocatechuic acid (PCA), likely to be the major competing pathway. The method of van der Geize [11] was used to generate a Δ*pcaHG* gene deletion mutant strain of *R. jostii* RHA1, using vector pK18*mobsacB*. The Δ*pcaHG* gene deletion strain showed only slightly reduced growth on lysogeny broth, compared to wild-type *R. jostii* RHA1, but when grown on M9 minimal media containing 0.1% PCA as carbon source, showed little or no growth over 48 h (see Additional file 1: Figure S3), consistent with the β-ketoadipate pathway being the major pathway for catabolism of protocatechuic acid. When grown on M9 minimal media containing 0.1% 4-hydroxybenzoic acid, metabolite analysis by HPLC showed the accumulation of PCA, consistent with the deletion of *pcaHG* genes (Additional file 1: Figure S4).

Expression plasmid pTipQC2 [9] was used to express the *Sphingobium* SYK-6 *ligAB* genes [12], or the *Paenibacillus* sp. JJ-1b *praA* gene [13], and these recombinant plasmids were transformed into the *R. jostii* Δ*pcaHG* strain. Growth of *R. jostii* Δ*pcaHG*/pTipQC2*ligAB* on M9 containing 0.1% 4-hydroxybenzoic acid and 0.4% glucose resulted in the production of 486 mg/L 2,4-PDCA after 144 h, compared with 90–125 mg/L for wild-type *R. jostii* RHA1 containing the same recombinant plasmid (see Additional file 1: Figure S5). Growth of *R. jostii* Δ*pcaHG*/pTipQC2*praA* on M9 containing 0.1% 4-hydroxybenzoic acid and 0.4% glucose resulted in the production of a higher titre of 810 mg/L 2,5-PDCA after 144 h (see Additional file 1: Figure S5). These titres correspond to 49% and 81% molar yield for biotransformation of 4-hydroxybenzoic acid to 2,4-PDCA and 2,5-PDCA respectively.

**Fig. 2:**
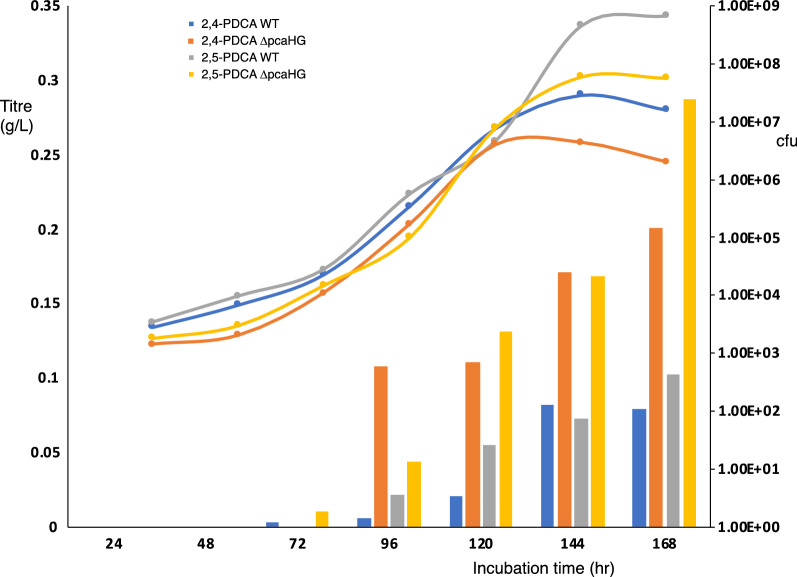
Growth curves (cfu, right hand scale) and titres of 2,4-PDCA production from 2 L bioreactor growths of *R. jostii* RHA1 wild-type (blue) and *R. jostii* D*pcaHG* containing pTipQC2ligAB (orange), and titres of 2,5-PDCA production by *R. jostii* wild-type (gray) and *R. jostii* D*pcaHG* containing pTipQC2praA (yellow), grown on M9 minimal media containing 1% wheat straw lignocellulose. Left hand scale shows titre (in g/L) of 2,4-PDCA or 2,5-PDCA product. Titres in g/L calculated from HPLC peak size, compared with authentic standards. HPLC traces are shown in Additional file 1: Figure S6. Data shown are averages of two measurements from a single experiment, and are representative of several experiments conducted similarly

Growth of *R. jostii* Δ*pcaHG*/pTipQC2*ligAB* on M9 minimal media containing 1% wheat straw lignocellulose resulted in the production of 200 mg/L 2,4-PDCA after 168 h (see Additional file 1: Figure S6), compared with 82 mg/L for wild-type *R. jostii* RHA1 containing the same recombinant plasmid. When grown in a 2 L bioreactor, optimum production was observed after 144–168 h growth (see Fig. 2). Growth of *R. jostii* Δ*pcaHG*/pTipQC2*praA* on M9 containing 1% wheat straw lignocellulose resulted in the production of 287 mg/L 2,5-PDCA after 168 h (see Additional file 1: Figure S6), compared with 102 mg/L for wild-type *R. jostii* RHA1 containing the same recombinant plasmid. Therefore, removal of the competing β-ketoadipate pathway by deletion of *pcaHG* genes gives a 2.5–threefold increase in the titre of PDCA bioproduct obtained from polymeric lignin.

However, during these experiments we found that significant loss of the pTipQC2 plasmid from recombinant *R. jostii* RHA1 strains was observed upon storage for > 2 weeks (see Additional file 1: Figure S7), perhaps due to recombination with chromosomal genes, or selective pressure for plasmid loss. Therefore, in order to generate a stable production strain, it was necessary to integrate the *ligAB* or *praA* genes onto the chromosome of *R. jostii* RHA1.

### Insertion of ligAB genes onto R. jostii RHA1 chromosome

**Fig. 3:**
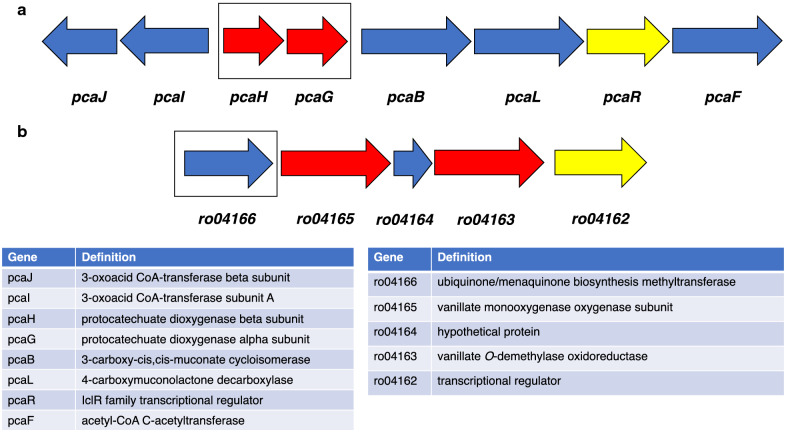
*R. jostii* RHA1 gene clusters into which *ligAB* or *praA* genes were inserted. The genes replaced by gene insertion are boxed. **a** Gene cluster encoding genes of the beta-ketoadipate pathway, in which *pcaHG* encode protocatechuate 3,4-dioxygenase; **b** gene cluster encoding vanillate mono-oxygenase, in which ro04166 encodes a methyltransferase of unknown function

Gene insertion of *ligAB* or *praA* genes onto the *R. jostii* RHA1 chromosome was carried out using homologous recombination. Since gene deletion of the *pcaHG* genes was shown to enhance PDCA titre, and since the β-ketoadipate pathway operon is known to be inducible by vanillic acid or 4-hydroxybenzoic acid [14, 15] which are intermediates in lignin breakdown, the first strategy was to insert the *Sphingobium* SYK-6 *ligAB* or *Paenibacillus* sp. JJ-1b *praA* genes in place of the *pcaHG* genes encoding the first enzymes of the β-ketoadipate pathway (see Fig. 3a). Vector pK18*mobsacB* was modified by addition of *ligAB* or *praA* genes and 1 kb genomic DNA sequences upstream and downstream of the *pcaHG* genes (see Additional file 1: Figure S8, S9). After transformation into *R. jostii* RHA1, kanamycin selection and sucrose counter-selection, the gene replacement mutants were isolated and confirmed by internal and external PCR reactions.

The *ligAB* or *praA* genes were also inserted via the same methodology into the gene cluster expressing the *vanAB* genes encoding vanillate demethylase, a cluster known to be strongly inducible by vanillin [16]. The *vdh* gene (ro02986) encoding vanillin dehydrogenase has previously been targeted for gene deletion, in order to generate vanillin, but is located in a different gene cluster [3]. The ro04166 gene which is annotated as a methyltransferase gene that is apparently not needed for degradation of vanillic acid, so was selected for replacement by *ligAB* or *praA* (see Fig. 3b).

The recombinant strains were first tested for their growth phenotypes on M9 minimal media containing different aromatic carbon sources. Whereas wild-type *R. jostii* RHA1 is able to grow on M9 containing 0.1% PCA or precursors vanillic acid or 4-hydroxybenzoic acid, mutants *pcaHG:ligAB* or *pcaHG:praA* were unable to grow on 0.1% vanillic acid or 0.1% 4-hydroxybenzoic acid, consistent with the β-ketoadipate pathway being the primary pathway for metabolism of these compounds. However, surprisingly, these mutant strains and the Δ*pcaHG* gene deletion strain were able to grow slowly on 0.1% PCA as carbon source (see Additional file 1: Figure S3), indicating that there is another pathway for metabolism of protocatechuic acid, that can be induced by the presence of higher concentrations of PCA. Mutants *ro01466:ligAB* and *ro01466:praA* were able to grow on M9 containing 0.1% PCA, vanillic acid or 4-hydroxybenzoic acid, consistent with metabolism of PCA via the β-ketoadipate pathway.

When the mutant strains containing *ligAB* or *praA* genes in place of the chromosomal *pcaHG* or *ro01466* genes were grown on minimal M9 media containing 0.1% vanillic acid, no production of 2,4-PDCA or 2,5-PDCA was observed. Although the *pca* gene cluster is inducible by vanillic acid, expression of the *ligAB* or *praA* genes in the absence of a dedicated gene promoter was found to be very low (Additional file 1: Figure S10), therefore, gene promoters were tested in order to achieve gene expression when inserted onto the *R. jostii* RHA1 chromosome.

### Testing of gene promoters for expression of ligAB genes in R. jostii RHA1

**Fig. 4:**
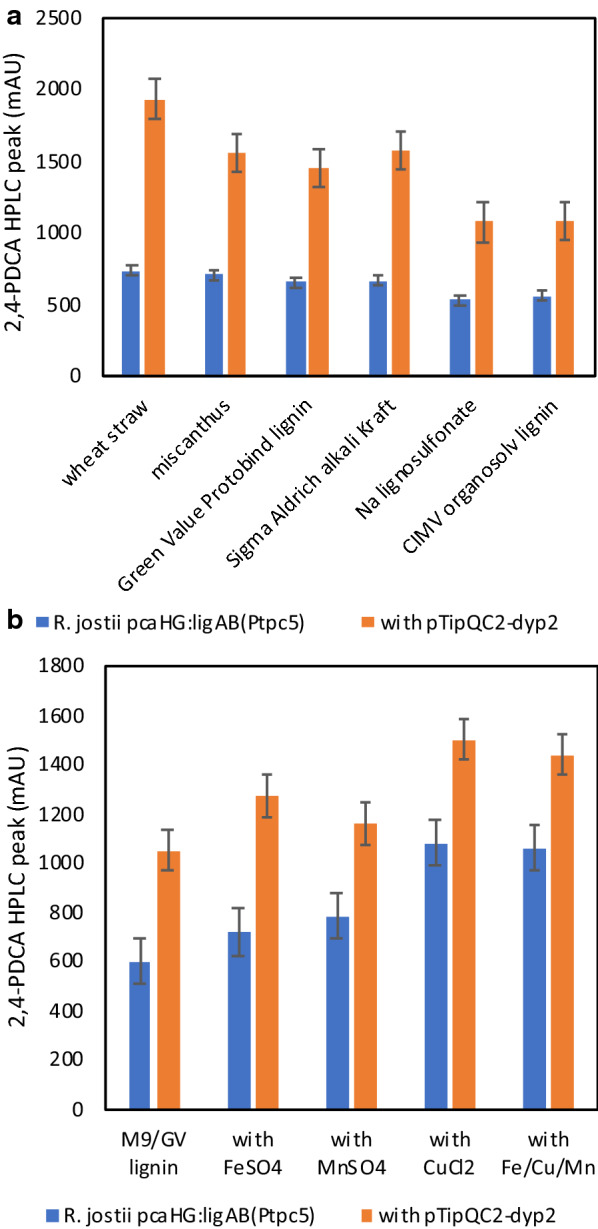
2,4-PDCA production (analysed by HPLC) for *R. jostii pcaHG*:*ligAB*(P_tpc5_) and *R. jostii pcaHG*:*ligAB*(P_tpc5_)/pTipQC2-dyp2 grown on 4 mL scale on M9 media containing different carbon sources and media additives. **a** Growth on M9 minimal media containing different lignocellulose or lignin feedstocks, with or without expression of *Amycolatopsis dyp2* gene, for 168 h at 30 °C. **b** Growth on M9 media containing 0.2% Green Value lignin with or without expression of *Amycolatopsis dyp2* gene, with addition of 0.15 g/L FeSO_4_, MnSO_4_, CuCl_2_, or all three salts, for 168 h at 30 °C. Error bars are standard deviations from triplicate biological replicates

Several promoters were tested for chromosomal gene expression of the *ligAB* genes (responsible for 2,4-PDCA production) inserted in place of *pcaHG* genes. New constructs containing promoters and *ligAB* genes were assembled using the knock-in vector as shown in Fig. 4. The P_icl_ promoter has been used previously in *R. erythropolis* [17], and is inducible with 1–5% methanol. The P_nitA_ promoter from *Rhodococcus rhodochrous* J1 [18] has been used previously for expression in several *Streptomyces* species [19], and is inducible by 0.1% ε-caprolactam [18, 19]. The P_bad_ promoter is commonly used in *Escherichia coli* [20], has been reported as an inducible expression promoter in *Rhodococcus opacus* [21], and is inducible with 0.1% arabinose. Also the P_tipA_ promoter from pTipQC2 was modified with 5 mutations in the -10 and -35 regions, as described by Nakashima and Tamura [9], resulting in a constitutive promoter, which we termed P_tpc5_ (see Additional file 1: Figure S11).

*R. jostii pcaHG:ligAB*(P_icl_), *R. jostii pcaHG:ligAB*(P_nitA_) and *R. jostii pcaHG:ligAB*(P_tpc5_) each showed LigAB enzyme activity in cell-free extract when grown in LB media using the appropriate inducer (shown for *R. jostii pcaHG:ligAB*(P_icl_) in Additional file 1: Figure S12), but no activity was observed using the P_bad_ promoter, inducing with 0.1% arabinose. Production of the LigAB proteins was observed by SDS-PAGE of cell extracts (see Additional file 1: Figure S13). LigAB activity in cell-free extracts was comparable to that obtained using the thiostrepton-inducible P_tipA_ promoter from pTipQC2 (see Additional file 1: Figure S14).

In order to verify the catalytic activity of the expressed LigAB enzyme, biotransformation of 0.05% PCA was carried out using *R. jostii pcaHG:ligAB*(P_icl_) and *pcaHG:ligAB*(P_tpc5_) whole cells grown previously in LB media. In a 30 min biotransformation, efficient conversion of PCA to 2,4-PDCA was observed (see Additional file 1: Figure S15), with 87% consumption of PCA using *R. jostii pcaHG:ligAB*(P_icl_) cells, and 98% consumption of PCA for *R. jostii pcaHG:ligAB*(P_tpc5_) cells.

### Production of 2,4-PDCA from polymeric lignin

Having established the conversion of protocatechuic acid to 2,4-PDCA, the production of 2,4-PDCA was first tested using an aromatic precursor 4-hydroxybenzoic acid as carbon source. Growth of *R. jostii pcaHG:ligAB*(P_tpc5_) in LB media containing 0.1% 4-hydroxybenzoic acid resulted in efficient conversion to 2,4-PDCA, but growth in M9 minimal media in the presence of 0.1% glucose gave very weak production of 2,4-PDCA, likely due to catabolite repression by glucose [22]. However, growth of this strain using 0.1% yeast extract as media additive in place of glucose was found to generate 2,4-PDCA and PCA from M9/0.1% 4-hydroxybenzoic acid (see Additional file 1: Figure S16).

Strains containing chromosomally inserted *ligAB* genes were then tested for production of 2,4-PDCA on M9 media containing Green Value Protobind lignin (GVPL), a commercially available soda lignin prepared from wheat straw/sarkanda. This lignin shows partial aqueous solubility which makes it more convenient for microbial transformation, and it has been structurally characterised as a S/G/H lignin containing predominantly β-O-4 units, M_W_ 3270 M_n_ 620 g/mol [23]. Growth of *R. jostii pcaHG:ligAB*(P_tpc5_) in M9/1% GVPL resulted in the formation of 2,4-PDCA and PCA after 168 h as observed by HPLC analysis (see Additional file 1: Figure S17).

In order to optimise the growth media, screening of media additives (1 g/L yeast extract, 0.15 g/L FeSO_4_, 0.5 g/L NH_4_Cl) was carried out in 4 mL incubations. Addition of 0.15 g/L FeSO_4_ was found to improve the titre of 2,4-PDCA observed using GVPL as carbon source (see Additional file 1: Figure S17), perhaps as a cofactor for host peroxidase enzymes responsible for lignin oxidation. At 50 mL scale, in the presence of 0.15 g/L FeSO_4_, *R. jostii pcaHG:ligAB*(P_tpc5_) formed 2,4-PDCA (164 mg/L) and PCA in titres of 164 mg/L and 200 mg/L respectively after 240 h at 30 °C from 1% GVPL as feedstock, and 290 mg/L 2,4-PDCA was formed using 1% wheat straw lignocellulose as feedstock, verifying that 2,4-PDCA could be formed from a polymeric lignin feedstock.

Constructs containing other gene promoters were then compared, in cultures grown in M9 containing either 1% wheat straw lignocellulose or 1% GVPL, for 168 h at 30 °C. Growth of *R. jostii pcaHG:ligAB*(P_icl_), with induction by 5% methanol, was found to generate 70 mg/L 2,4-PDCA using 1% GVPL (see Additional file 1: Figure S17), but no 2,4-PDCA product was observed using 1% wheat straw lignocellulose as feedstock. Growth of *R. jostii pcaHG:ligAB*(P_nitA_), with induction by 0.1% ε-caprolactam, was found to generate 100 mg/L 2,4-PDCA using 1% GVPL, and 79 mg/L 2,4-PDCA using 1% wheat straw lignocellulose. These data establish that the P_tpc5_, P_icl_, and P_nitA_ promoters are functional for *ligAB* expression in *R. jostii* RHA1. Although promoter P_icl_ gave the most efficient conversion of PCA to 2,4-PDCA, catalysed by LigAB, constitutive promoter P_tpc5_ gave highest titres of the desired 2,4-PDCA product from lignin or lignocellulose feedstocks.

### Enhancement in 2,4-PDCA production by expression of lignin-oxidising enzyme

We then investigated whether the titre of 2,4-PDCA products formed from polymeric lignin feedstock could be enhanced by increasing the rate of lignin oxidation, by overexpression of *Amycolatopsis* sp. 75iv2 Dyp2 peroxidase, reported to have high manganese peroxidase activity for lignin oxidation [24]. The *Amycolatopsis dyp2* gene was overexpressed in *R. jostii pcaHG:ligAB*(P_tpc5_) using the pTipQC2 expression plasmid, and production of the Dyp2 enzyme was observed in cell extracts by SDS-PAGE (Additional file 1: Figure S18). *R. jostii pcaHG:ligAB*(P_tpc5_) with and without pTipQC2-dyp2 were grown on M9 minimal media containing 1% wheat straw lignocellulose for 120 h at 30 °C, and 1.6-fold enhancement of 2,4-PDCA production was observed (186 mg/L with pTipQC2-dyp2, 116 mg/L without pTipQC2-dyp2, see Additional file 1: Figure S19).

The *R. jostii pcaHG:ligAB*(P_tpc5_) strain with and without pTipQC2-dyp2 was then tested at small scale in M9 minimal media containing a wider range of lignocellulose and polymeric lignin feedstocks at 1% (w/v) concentration. As shown in Fig. 4a, highest concentration of 2,4-PDCA was observed using wheat straw lignocellulose, but efficient production of 2,4-PDCA was also observed using miscanthus lignocellulose, Green Value protobind lignin, and alkali Kraft lignin (Sigma-Aldrich), and slightly lower levels of production were observed using organosolv lignin or industrial lignosulfonate. In each case overexpression of *dyp2* gave increases in titre of 60–160% on a small scale.

Testing the addition of different concentrations of Fe^2+^, Cu^2+^ and Mn^2+^ salts to cultures of *R. jostii pcaHG:ligAB*(P_tpc5_) grown in M9 media containing 0.2% GVPL on a small scale (4 mL) revealed that 20–40% enhancement in 2,4-PDCA titre could be achieved by inclusion of metal ions in the media, as shown in Fig. 4b, with highest activity observed in the presence of 0.15 g/L CuCl_2_, and overexpression of *dyp2* gave increases of 35–75% in titre on a small scale. Small amounts of PCA product were also observed by HPLC analysis (see Additional file 1: Figure S20).

**Fig. 5:**
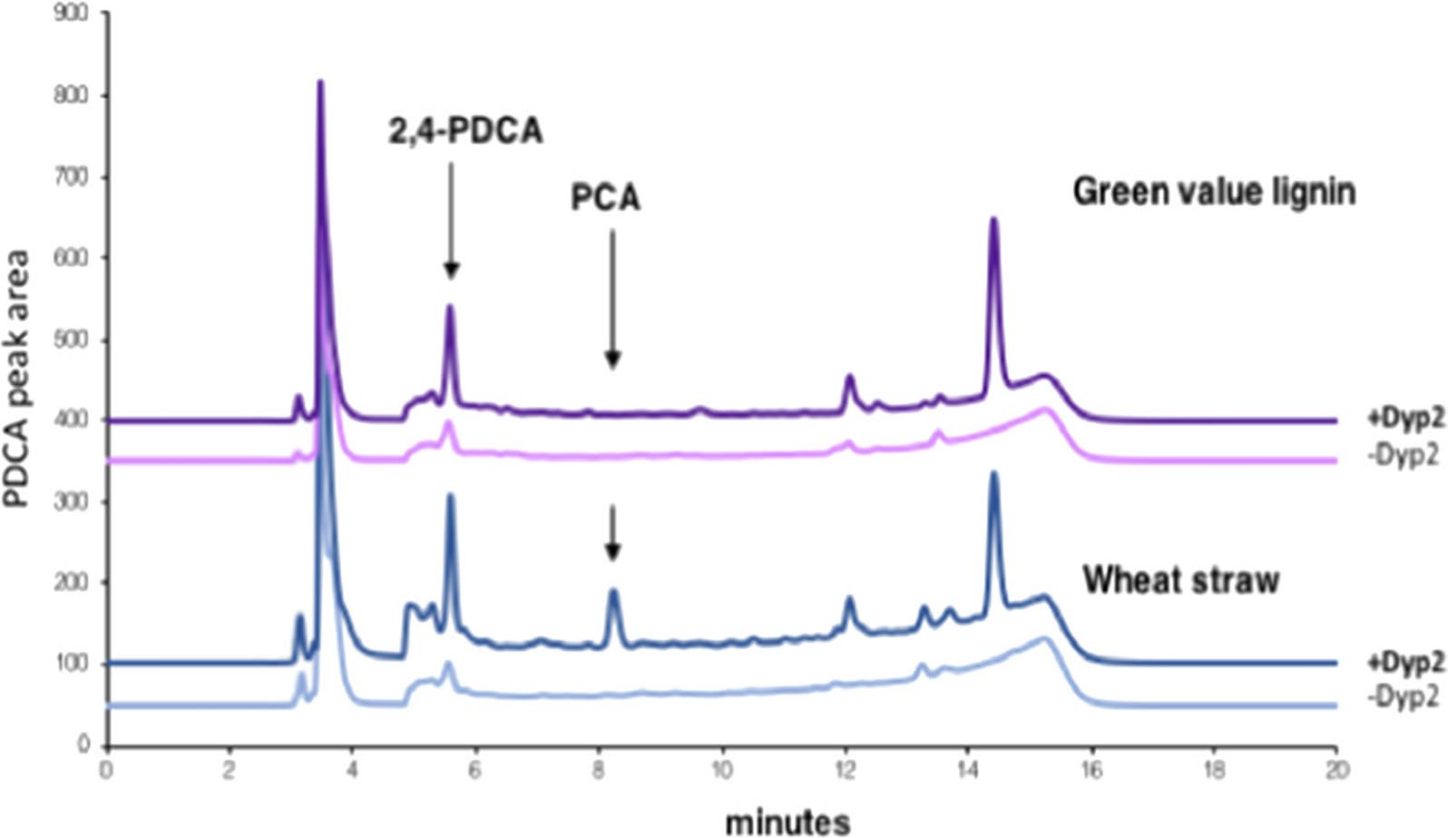
2,4-PDCA production by *R. jostii pcaHG*:*ligAB*(P_tpc5_) construct with or without expression of *Amycolatopsis* sp. 75iv2 *dyp2* gene in 2.5 L bioreactor after 40 h, analysed by reverse phase HPLC. Maximum product titre was observed at 40 h for strain expressing *dyp2*, whereas strain lacking *dyp2* showed maximum titre after 168 h (see Table 1). PCA, protocatechuic acid

Growth of the *R. jostii pcaHG:ligAB*(P_tpc5_) and *R. jostii pcaHG:ligAB*(P_tpc5_)/pTipQC2-dyp2 strains was then scaled up to a 2.5L bioreactor, and grown on minimal media containing 1% wheat straw or Green Value Protobind lignin feedstocks, monitoring the time-course of 2,4-PDCA production. It was found that overexpression of *dyp2* resulted in faster release of PDCA product as well as improved PDCA titre (see Fig. 5, Additional file 1: Figure S21), with maximal PDCA production observed after 40 h, as opposed to 168 h for the previously published plasmid-based construct [8].

Recovery of the 2,4-PDCA bioproduct was previously reported via anion exchange [8], however, this method gave an aqueous solution of PDCA product, rather than a solid product. It was found that the 2,4-PDCA product could be extracted into isopropanol after addition of 30% (w/v) NaCl to the culture broth (see Additional file 1: Figure S22), and could then be isolated as a solid by evaporation. The isolated product was found to show the expected HPLC retention time (see Additional file 1: Figure S23) and UV–vis absorption at 275 nm, and the expected ^1^H NMR signals (see Additional file 1: Figure S24). Following this protocol, the isolated titre of 2,4-PDCA by *R. jostii pcaHG:ligAB*(P_tpc5_)/pTipQC2-dyp2 from M9/1% wheat straw lignocellulose was 330 mg/L, from a 40 h bioconversion. The higher titres obtained by this method we believe are due to the binding of some PDCA product to hydrophobic lignin or lignocellulose particles, hence the estimates obtained from the earlier small scale HPLC analyses may have under-estimated the amount of product formed. The titres obtained in the course of this work are summarised in Table 1.

**Table 1:** PDCA titres (in mg/L) from (A) wild-type *R. jostii* RHA1 containing plasmid-expressed *ligAB* or *praA* genes [8]; (B) Δ*pcaHG* gene deletion strain containing plasmid-expressed *ligAB* or *praA* gene; (C) chromosomally expressed *ligAB* or *praA* genes under promoter control

Strain	Product	Time (h)	Carbon source for M9 minimal media (mg/L)		
			0.1% VA or 4-HBA	1% wheat straw	1% GV lignin
A. Wild-type strain/plasmid					
* R. jostii* pTipQC2-*ligAB*	2,4-PDCA	168	112^a^	90–125	
* R. jostii* pTipQC2-*praA*	2,5-PDCA	168	80^a^	65–106	
B. Gene deletion/plasmid					
* R. jostii* Δ*pcaHG*/pTipQC2-*ligAB*	2,4-PDCA	168	486^b^	200	
* R. jostii* Δ*pcaHG*/pTipQC2-*praA*	2,5-PDCA	168	810^b^	287	
C. Chromosomal expression					
* R. jostii* Δ*pcaHG*:*ligAB* (no promoter)	2,4-PDCA	168	ND^b^	ND	
* R. jostii* Δ*pcaHG*:*ligAB(P*_*nitA*_*)*	2,4-PDCA	168		79	100
* R. jostii* Δ*pcaHG*:*ligAB(P*_*icl*_*)*	2,4-PDCA	168		ND	70
* R. jostii* Δ*pcaHG*:*ligAB(P*_*tpc5*_*)*	2,4-PDCA	168		290	164
* R. jostii* Δ*pcaHG*:*ligAB(P*_*tpc5*_*)/pTipQC2-dyp2*	2,4-PDCA	40		330	240

Data shown are averages of measurements from two biological replicates. Key: a, vanillic acid (VA); b, 4-hydroxybenzoic acid (4-HBA); GV lignin, Green Value Protobind lignin; ND, product not detected

## Discussion

In our previous study, 2,4-PDCA or 2,5-PDCA were generated as bio-products from lignin degradation by *R. jostii* RHA1 strains containing either *ligAB* or *praA* genes overexpressed on the pTipQC2 plasmid [8]. The recombinant strains used in the previous study had some limitations for scale-up: (1) the strains required an expensive inducer (thiostrepton) and antibiotic; (2) the growth time was very long (168–216 h); and (3) the plasmid-borne genes were subsequently found to be somewhat unstable in *R. jostii* RHA1. Therefore, the aims of this study were to insert the *ligAB* or *praA* genes onto the *R. jostii* RHA1 chromosome, achieve efficient gene expression, and optimise PDCA production.

Gene deletion of the *pcaHG* genes initiating the competing β-ketoadipate pathway was found to result in significant enhancements in PDCA titre, 4–eightfold from aromatic carbon sources, and 2–threefold from lignocellulose as carbon source. Interestingly, the Δ*pcaHG* gene deletion strain was still able to grow weakly on M9/PCA, indicating the presence of another competing pathway from PCA in *R. jostii* RHA1. In a separate study, we have recently identified the genes responsible for a pathway in *R. jostii* RHA1 and *Agrobacterium* sp. proceeding via conversion of protocatechuic acid to hydroxyquinol [25].

Three promoters have been shown to be functional for expression of *ligAB* genes integrated onto the *R. jostii* RHA1 chromosome, of which P_tpc5_ is constitutive, and P_icl_ and P_nitA_ can be activated by inexpensive inducers methanol and ε-caprolactam respectively. These constructs generate 2,4-PDCA product using either wheat straw lignocellulose or a commercially available soda lignin as carbon source. 2,4-PDCA production from lignin feedstocks was enhanced by overexpression of the *Amycolatopsis* sp. 75iv2 *dyp2* gene, and inclusion of Fe^2+^, Mn^2+^ and Cu^+^ salts in the media further enhances 2,4-PDCA production, presumably by activating lignin-oxidising Dyp-type peroxidase [24, 26] and multi-copper oxidase enzymes. Overexpression of the *Amycolatopsis dyp2* gene in the optimised production strain also reduces the time taken to reach maximum titre of 2,4-PDCA, from 168 h to 40 h. The productivity of 2,4-PDCA production from lignocellulose in the optimised production strain has therefore been improved > tenfold from 0.5–0.7 mg/L/h for wild-type *R. jostii* pTipQC2-*ligAB* [8] to 8.25 mg L^−1^ h^−1^ for *R. jostii* Δ*pcaHG*:*ligAB(P*_*tpc5*_*)/*pTipQC2-dyp2. These two effects of *dyp2* overexpression demonstrate that the rate of lignin depolymerisation is a limiting factor in bioproduct formation. Overexpression of lignin-oxidising multi-copper oxidase enzyme SLAC in *Amycolatopsis* sp. 75iv3 has been reported by Singh et al., leading to increased acid-precipitable lignin formation and enhanced production of monocyclic aromatic compounds [27]. The titre of 330 mg/L 2,4-PDCA from 1% wheat straw lignocellulose corresponds to a conversion yield of approximately 16% of the lignin fraction present. Further improvements in yield are likely to require improved knowledge of the biochemical processes involved in lignin depolymerisation and uptake of lignin fragments.

## Conclusion

The production of 2,4-PDCA using an engineered *R. jostii* RHA1 strain from a commercially available lignin feedstock is a significant step towards the generation of bioproducts from lignin or lignocellulose feedstocks. Literature studies on bioproduct generation from lignin using *Pseudomonas putida* KT2440 or *Corynebacterium glutamicum* have used biomass pretreated by alkali [4–6] or supercritical water [7], containing predominantly low molecular weight aromatic compounds, whereas lignin streams produced from pulp/paper manufacture or cellulosic biofuel production are polymeric. The ability of engineered *R. jostii* RHA1 to utilise polymeric lignin as feedstock allows the use of industrial lignins to generate 2,4-pyridinedicarboxylic acid. We have shown that commercially available Green Value Protobind soda lignin is an effective feedstock for *R. jostii* RHA1 bioconversions. Moreover, the ability of engineered *R. jostii* RHA1 strains to utilise Kraft lignin as carbon source is remarkable, since Kraft lignin has a condensed structure that is generally found to be more difficult to valorise [10]. The ability to utilise Kraft lignin was also noted in our earlier study [8], confirmed here, and implies that *Rhodococcus jostii* RHA1 is able to break down condensed as well as uncondensed polymeric lignins.

Metabolic engineering for lignin degradation is still an emerging field, due to the limited number of microbial hosts available for lignin degradation, and the genetic tools available for those organisms. *Rhodococcus jostii* RHA1 [3, 8] and *Pseudomonas putida* KT2440 [4, 6] have both been used successfully as microbial hosts for lignin bioconversion to bioproducts, due to their activity for lignin depolymerisation [28], a property not shared by *Escherichia coli* K12. *Corynebacterium glutamicum* [7] and *Rhodococcus opacus* [29] have also been reported as microbial hosts for bioproduct formation from lignin. An engineered strain of *P. putida* KT2440 has also been reported to produce 2,5-PDCA bioproducts, using 4-hydroxybenzoic acid or glucose as feedstocks [30]. The ability of engineered *Rhodococcus jostii* RHA1 strains to generate 2,4-PDCA bio-products from a range of lignin feedstocks makes this host well-suited to convert industrial lignins or lignocellulose to bioplastic monomers.

## Methods

### Bacterial strains and chemicals

All chemicals were purchased form Sigma Aldrich unless otherwise stated. Green Value Protobind 1000 soda lignin was purchased from Green Value SA (Orbe, Switzerland); Na lignosulfonate was a gift from Borregaard LignoTech (Sarpsborg, Norway); wheat straw organosolv lignin was a gift from CIMV (Levallois Perret, France).

*Rhodococcus jostii* RHA1 was used as the ancestral strain. For routine growth and maintenance, *R. jostii* RHA1 cells were cultured in liquid or solid lysogeny broth (LB) medium, with the appropriate selection medium at 30 °C and with shaking at 180 rpm if required.

### Construction of the *Rhodococcus jostii* RHA1 markerless pcaHG deletion mutant (ΔpcaHG)

The Δ*pcaHG* gene deletion mutant was made using the pk18*mobsacB* plasmid, which uses *sacB* (confers sucrose sensitivity) as a counter-selectable marker [11]. PCR was used to amplify two 1 kb regions of chromosomal DNA on either side of the *pcaH* and *pcaG* genes. The PCR products included restriction sites for cloning into the pK18*mobsacB* plasmid. The downstream 1 kb region included the restriction sites *Xba*I and *Pst*I, while the upstream 1 kb region included the restriction sites *Pst*I and *Hind*III. All three products were ligated together and the resulting construct confirmed by sequencing and restriction digestion (see Additional file 1: Figure S1). The recombinant plasmid was taken up into *R. jostii* RHA1 by electroporation (see below), and recombinant colonies selected by kanamycin resistance. Isolation of the double cross-over gene deletion was carried out using sucrose resistance counter-selection [11], and the Δ*pcaHG* markerless deletion was confirmed by PCR (Additional file 1: Figure S2).

### Plasmid expression of recombinant genes

The thiostrepton inducible expression vector PTip-QC2 [9] was used for expression of *Sphingobium SYK-6 ligAB* encoding protocatechuate 4,5-dioxygenase [12], *Paenibacillus* sp. JJ-1b *praA* encoding protocatechuate 2,3-dioxygenase [13], or *Amycolatopsis *sp.* dyp2* [24] in *R. jostii* RHA1, as described previously [8].

### Generation of *R. jostii* RHA1 knock-in mutants and chromosomal expression

For chromosomal expression of the *S. paucimobilis* SYK-6 *ligAB* dioxygenase gene, a suicide vector was constructed based upon pUC19, containing an apramycin resistance cassette and the *ligAB* dioxygenase gene with or without promoter, flanked by 1 kb of homologous DNA regions upstream and downstream of the integration site (see Additional file 1: Figures S8, S9). The knock-in construct was introduced into *R. jostii* RHA1 by electroporation. Successful removal of the *pcaH* and *pcaG* genes was confirmed by PCR.

Constructs containing the *ligAB* genes under the control of P_icl_, P_nitA_ or P_tpc5_ promoters were grown on M9 minimal media containing carbon sources as indicated in the text, for 168 h at 30 °C. The P_icl_ promoter was induced by addition of 5% (v/v) methanol after 24 h, and further additions of 5% methanol made after each 24 h. The P_nitA_ promoter was induced by addition of 0.1% (w/v) ε-caprolactam after 24 h.

### Electroporation of *R. jostii* RHA1

*Rhodococcus jostii* RHA1 was transformed with exogenous DNA using electroporation. A single colony of *R. jostii* RHA1 was used to inoculate 10 ml of sterile lysogeny broth (LB) and grown overnight at 30 °C with shaking at 180 rpm. This overnight culture was used to inoculate 50 ml of lysogeny broth (LB, 10 g tryptone, 10 g sodium chloride, 5 g yeast extract per litre) which was then grown overnight at 30 °C, with shaking at 180 rpm. The cells were harvested using centrifugation (5000*g*) at 4 °C and the pelleted cells were then washed three times with sterile ice-cold 10% (v/v) glycerol at 4 °C. Following the final glycerol wash, the pelleted cells were re-suspended in the residual 10% glycerol and stored at − 80 °C. For the electroporation, 80 μL of cells was used for each transformation, with 3 μL of plasmid DNA. The electroporation was performed on ice, using 2 mm electroporation cuvettes, with the following conditions; 2.5 kV, 25 μF and 400 Ω. After electroporation, 1 mL of sterile ice-cold LB was added to the electroporation cuvette and incubated without shaking overnight at 30 °C. Following overnight incubation, 200 μL of cell culture was plated out onto LB plates with the appropriate selection and incubated at 30 °C. Colonies were usually visible after 2–3 days.

### Growth of bacterial cultures

The growth of wild-type *R. jostii* RHA1 strain and the Δ*pcaHG* deletion strain were compared. For growth rates, cultures were grown in either LB or M9 minimal medium with 0.1% (w/v) PCA as the sole carbon source. Single colonies were used to inoculate 10 mL of LB, which were grown overnight at 30 °C at 180 rpm. For growth in the minimal medium, the cells were pelleted using centrifugation and washed twice in sterile M9. 5 μL of the overnight culture was then used to inoculate 200 μL of culture in a 96-well deep-well plate containing M9 media (4 mL per well) with media additives as described in the text. The plates were then incubated at 30 °C for 48 h, and A_600_ measured.

### Metabolite analysis

For metabolite analysis, 500 μL aliquots of culture were removed and combined with 500 μL of HPLC grade methanol/0.1% trifluoracetic acid. Samples were vortexed and then centrifuged (microcentrifuge) for 15 min. HPLC analysis of the supernatant was performed using a Zorbax Eclipse plus (Agilent) C_18_ reverse phase HPLC column. The HPLC solvents were water/0.1% trifluoracetic acid (solvent A) and methanol/0.1% trifluoracetic acid (solvent B). The applied gradient was 15% B for 15 min; 15–50% B over 1 min; 50–15% B over 8 min, at a flow rate of 0.8 mL/min. UV detection was at 270 nm. Analyses of 2,4-PDCA and 2,5-PDCA were compared with authentic standards of 2,4-PDCA and 2,5-PDCA (from Sigma-Aldrich), with detection at 270 nm. Product titres were determined at 4 mL scale (deep well microtitre plate) using triplicate biological replicates, and at 50–100 mL scale (shake flask) using duplicate biological replicates.

### Bioreactor microbial biotransformation

Cultures of *R. jostii* RHA1 maintaining pTipQC2-*ligAB* or pTipQC2-*praA* were grown at 30 °C in 2 L of M9 minimal media (6 g/L Na_2_HPO_4_, 3 g/L KH_2_PO_4_, 0.5 g/L NaCl, 1.0 g/L NH_4_Cl, 2 mM MgSO_4_, 0.5 mM CaCl_2_) with 1 ml of trace elements (FeSO_4_.7H_2_O, 0.5 g; ZnSO_4_.7H_2_O, 0.4 g; MnSO_4_.H_2_O, 0.02 g; H_3_BO_3_, 0.015 g; NiCl_2_.6H_2_O, 0.0g/L; EDTA, 0.25 g; CoCl_2_.6H_2_O, 0.05 g; CuCl_2_.2H_2_O, 0.005 g; NaMoO_4_.2H_2_O, 2.0 g; and Na-EDTA, 5.0 g made up to 1 L with dH_2_O) in an Electrolab FerMac 3010 bioreactor. Wheat straw lignocellulose pellets were added (final concentration 1% (w/v)) and yeast extract (0.25%). Chloramphenicol was added to 50 μg/mL and cultures were induced by addition of 5 μg/mL thiostrepton after 24 h. The culture was then grown for 40–168 h at 30 °C, supplementing with 5 μg/mL thiostrepton every 48 h. Samples were removed ascetically for HPLC analysis and estimation of cell growth from colony forming units.

The culture medium was centrifuged (5000*g*, 10 min). To the culture supernatant was added 30% (w/v) NaCl and 1 volume isopropanol, and the resulting mixture was stirred, forming two layers. The upper organic phase was separated, and an aliquot removed for HPLC analysis. Isopropanol was removed via rotary evaporation at reduced pressure, and residual water was then removed by freeze-drying, to give a solid residue, which was analysed by HPLC and ^1^H NMR spectroscopy.

### Assay of LigAB or PraA protocatechuate dioxygenase activity

Recombinant *R. jostii* RHA1 cells were collected by centrifugation (5000*g*) and washed three times with 5 mL of ice cold NaCl (150 mM). After the washes, the pelleted cells were resuspended into 500 μL of ice cold NaCl (150 mM) to which 500 μL of 0.1 mm/100 μm glass beads (Sigma-Aldrich) were added. The cells were then incubated on ice for 30 min before cell disruption by vigorous vortexing for 1 min followed by 1 min on ice, which was repeated 10 times. The lysed cells were then centrifuged for 1 min at 13,000*g* to pellet the cell debris and glass beads. 50 μL of the supernatant was then added to 950 μL of 0.4 mM PCA in 20 mM Tris buffer pH 8.0. The appearance of a visible yellow colour indicated dioxygenase activity, which was measured at 350 nm for PraA and 410 nm for LigAB [8], and normalised to the sample protein concentration for comparison.

## Supplementary Information

**Additional file 1:** **Figure S1**. Vector for construction of the Δ*pcaHG* markerless deletion *R. jostii* strain; **Figure S2**. PCR confirmation of the Δ*pcaHG* markerless deletion; **Figure S3**. Growth characteristics of Δ*pcaHG* markerless deletion and wild-type *R. jostii* strains; **Figure S4**. HPLC analysis of Δ*pcaHG* markerless deletion and wild-type *R. jostii* strains grown in M9 minimal media containing 0.1% 4-hydroxybenzoic acid; **Figure S5**. Production of 2,4-PDCA observed by HPLC analysis in *R. jostii* Δ*pcaHG* containing pTipQC2ligAB; **Figures S6**. HPLC traces for production of 2,4-PDCA by *R. jostii* Δ*pcaHG* containing pTipQC2ligAB, and for production of 2,5-PDCA by *R. jostii* Δ*pcaHG* containing pTipQC2praA; **Figure S7**. Agarose gels showing loss of plasmid DNA from *R. jostii* Δ*pcaHG* containing pTipQC2ligAB; **Figures S8,S9**. Chromosomal integration vector (S8) for insertion of ligAB or praA genes onto *R. jostii* chromosome, and relevant PCR primers (S9); **Figure S10**. Gene expression for praA and ligAB genes for chromosomal gene insertion constructs (without promoter) by RT-PCR; **Figure S11**. Nucleotide sequence of constitutive promoter P_tpc5_; **Figures S12,S13**. LigAB activity (S12) and protein production (S13) from pcaHG:ligAB(P_icl_) construct, induced with 1-8% methanol; **Figure S14**. LigAB activity observed using *R. jostii* p*pcaHG:ligAB* constructs containing four different promoters, grown in LB media; **Figure S15**. Whole cell biotransformation of protocatechuic acid (PCA) to 2,4-PDCA using *R. jostii* *pcaHG:ligAB*(P_icl_) and *R. jostii* *pcaHG:ligAB*(P_tpc5_); **Figure S16**. Production of 2,4-PDCA using *R. jostii* *pcaHG:ligAB*(P_tpc5_) grown on M9 minimal media containing 0.1% 4-hydroxybenzoic acid and 0.1% yeast extract, analysed by HPLC; **Figure S17**. Production of 2,4-PDCA from minimal media containing Green Value Protobind lignin by constructs containing chromosomal expression of ligAB genes, analysed by HPLC; **Figure S18**. SDS-PAGE gel of cell extracts of *R. jostii* *pcaHG:ligAB*(P_tpc5_) with or without pTipQC2-dyp2, showing expression of recombinant Dyp2 peroxidase; **Figure S19**. Production of 2,4-PDCA by *R. jostii* *pcaHG:ligAB*(P_tpc5_) with or without pTipQC2-dyp2, grown on M9 minimal media containing 1% wheat straw lignocellulose; **Figure S20**. Small scale (4 mL) testing of the production of 2,4-PDCA and protocatchuic acid (PCA) by *R. jostii* **pcaHG:ligAB**(P_tpc5_) with or without expression of Amycolatopsis dyp2 gene; **Figure S21**. Titre of 2,4-PDCA vs time from 2.5L bioreactor for *R. jostii* *pcaHG:ligAB*(P_tpc5_) grown on M9 minimal media containing 1% Green Value Protobind lignin at 30 °C;** Figures S22–S24**. Isolation of 2,4-PDCA product from fermentation broth: S22, Extraction of 2,4-PDCA into isopropanol; S23, HPLC analysis of extracted product; S24, ^1^H NMR analysis of isolated product.

## Data Availability

All data generated or analysed during this study are included in this published article and its additional file.
