# Supplementary material for: Metabolic engineering of Rhodococcus jostii RHA1 for production of pyridine-dicarboxylic acids from lignin
Source: Microb Cell Fact. 2021 Jan 19;20:15. doi: 10.1186/s12934-020-01504-z (PMC7814577; doi:10.1186/s12934-020-01504-z)
Supplement: Supplementary file 1 — Additional file 1: Figure S1. Vector for construction of the ΔpcaHG markerless deletion R. jostii strain; Figure S2. PCR confirmation of the ΔpcaHG markerless deletion; Figure S3. Growth characteristics of ΔpcaHG markerless deletion and wild-type R. jostii strains; Figure S4. HPLC analysis of ΔpcaHG markerless deletion and wild-type R. jostii strains grown in M9 minimal media containing 0.1% 4-hydroxybenzoic acid; Figure S5. Production of 2,4-PDCA observed by HPLC analysis in R. jostii ΔpcaHG containing pTipQC2ligAB; Figures S6. HPLC traces for production of 2,4-PDCA by R. jostii ΔpcaHG containing pTipQC2ligAB, and for production of 2,5-PDCA by R. jostii ΔpcaHG containing pTipQC2praA; Figure S7. Agarose gels showing loss of plasmid DNA from R. jostii ΔpcaHG containing pTipQC2ligAB; Figures S8,S9. Chromosomal integration vector (S8) for insertion of ligAB or praA genes onto R. jostii chromosome, and relevant PCR primers (S9); Figure S10. Gene expression for praA and ligAB genes for chromosomal gene insertion constructs (without promoter) by RT-PCR; Figure S11. Nucleotide sequence of constitutive promoter Ptpc5; Figures S12,S13. LigAB activity (S12) and protein production (S13) from pcaHG:ligAB(Picl) construct, induced with 1-8% methanol; Figure S14. LigAB activity observed using R. jostii ppcaHG:ligAB constructs containing four different promoters, grown in LB media; Figure S15. Whole cell biotransformation of protocatechuic acid (PCA) to 2,4-PDCA using R. jostii pcaHG:ligAB(Picl) and R. jostii pcaHG:ligAB(Ptpc5); Figure S16. Production of 2,4-PDCA using R. jostii pcaHG:ligAB(Ptpc5) grown on M9 minimal media containing 0.1% 4-hydroxybenzoic acid and 0.1% yeast extract, analysed by HPLC; Figure S17. Production of 2,4-PDCA from minimal media containing Green Value Protobind lignin by constructs containing chromosomal expression of ligAB genes, analysed by HPLC; Figure S18. SDS-PAGE gel of cell extracts of R. jostii pcaHG:ligAB(Ptpc5) with or without pTipQC2-dyp2, showi [file 12934_2020_1504_MOESM1_ESM.docx]

**Metabolic engineering of *Rhodococcus jostii* RHA1 for production of pyridine-dicarboxylic acids from lignin**

**Edward M. Spence, Leo Calvo-Bado, Paul Mines, and Timothy D.H. Bugg**

**Supporting Information**

**Contents**

Figure S1. Vector for construction of the Δ*pcaHG* markerless deletion *R. jostii* strain

Figure S2. PCR confirmation of the Δ*pcaHG* markerless deletion

Figure S3. Growth characteristics of Δ*pcaHG* markerless deletion and wild-type *R. jostii* strains

Figure S4. HPLC analysis of Δ*pcaHG* markerless deletion and wild-type *R. jostii* strains grown in M9 minimal media containing 0.1% 4-hydroxybenzoic acid

Figure S5. Production of 2,4-PDCA observed by HPLC analysis in *R. jostii* Δ*pcaHG* containing pTipQC2ligAB

Figures S6. HPLC traces for production of 2,4-PDCA by *R. jostii* Δ*pcaHG* containing pTipQC2ligAB, and for production of 2,5-PDCA by *R. jostii* Δ*pcaHG* containing pTipQC2praA.

Figure S7. Agarose gels showing loss of plasmid DNA from *R. jostii* Δ*pcaHG* containing pTipQC2ligAB

Figures S8,S9. Chromosomal integration vector (S8) for insertion of *ligAB* or *praA* genes onto *R. jostii* chromosome, and relevant PCR primers (S9)

Figure S10. Gene expression for *praA* and *ligAB* genes for chromosomal gene insertion constructs (without promoter) by RT-PCR

Figure S11. Nucleotide sequence of constitutive promoter P_tpc5_

Figures S12,S13. LigAB activity (S12) and protein production (S13) from *pcaHG*:*ligAB*(P_icl_) construct, induced with 1-8% methanol

Figure S14. LigAB activity observed using *R. jostii* *pcaHG*:*ligAB* constructs containing four

different promoters, grown in LB media

Figure S15. Whole cell biotransformation of protocatechuic acid (PCA) to 2,4-PDCA using *R. jostii* *pcaHG*:*ligAB(P_icl_)* and *R. jostii* *pcaHG*:*ligAB(P_tpc5_)*.

Figure S16. Production of 2,4-PDCA using *R. jostii* *pcaHG*:*ligAB(P_tpc5_)* grown on M9 minimal media containing 0.1% 4-hydroxybenzoic acid and 0.1% yeast extract, analysed by HPLC.

Figure S17. Production of 2,4-PDCA from minimal media containing Green Value Protobind lignin by constructs containing chromosomal expression of *ligAB* genes, analysed by HPLC.

Figure S18. SDS-PAGE gel of cell extracts of *R. jostii* *pcaHG*:*ligAB*(P_tpc5_) with or without pTipQC2-dyp2, showing expression of recombinant Dyp2 peroxidase

Figure S19. Production of 2,4-PDCA by *R. jostii* *pcaHG*:*ligAB*(P_tpc5_) with or without pTipQC2-dyp2, grown on M9 minimal media containing 1% wheat straw lignocellulose

Figure S20. Small scale (4 mL) testing of the production of 2,4-PDCA and protocatchuic acid (PCA) by *R. jostii* *pcaHG*:*ligAB*(P_tpc5_) with or without expression of *Amycolatopsis* *dyp2* gene.

Figure S21. Titre of 2,4-PDCA vs time from 2.5L bioreactor for *R. jostii* *pcaHG*:*ligAB*(P_tpc5_) grown on M9 minimal media containing 1% Green Value Protobind lignin at 30 ^o^C

Figures S22-S24. Isolation of 2,4-PDCA product from fermentation broth: S22, Extraction of 2,4-PDCA into isopropanol; S23, HPLC analysis of extracted product; S24, ^1^H NMR analysis of isolated product.


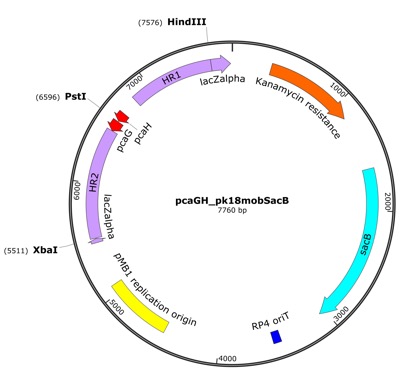


Figure S1. Vector for construction of the Δ*pcaHG* markerless deletion *R. jostii* RHA1 strain, based upon vector pK18mobsacB (van der Geize et al, 2001)


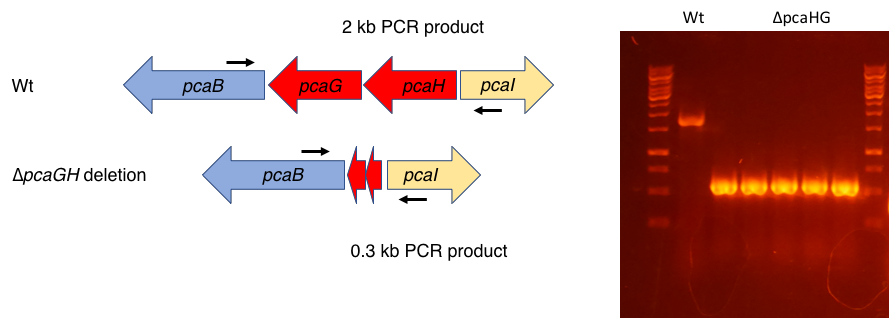


Figure S2. PCR confirmation of the Δ*pcaHG* markerless deletion


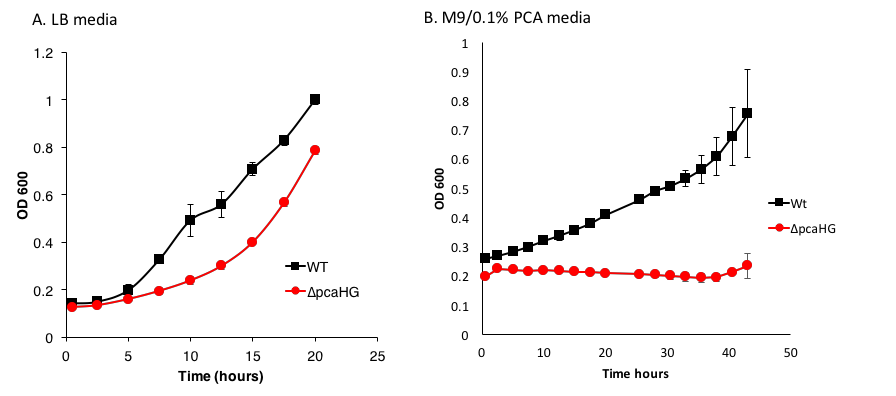


Figure S3. Growth characteristics of Δ*pcaHG* markerless deletion and wild-type *R. jostii* RHA1 strains in (A) LB broth (B) M9 minimal media containing 0.1% protocatechuic acid as sole carbon source, grown at 30 ^o^C. Error bars indicate the standard deviation from duplicate biological replicates.


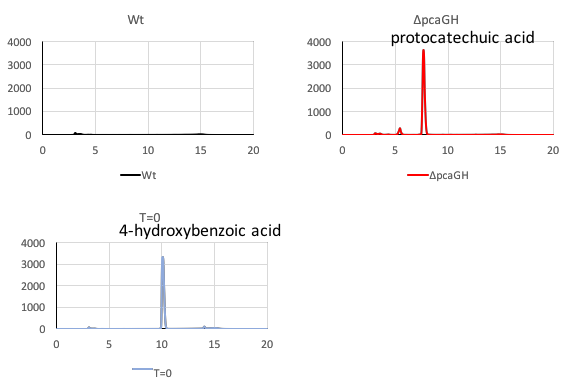


**C**

**B**

**A**

Figure S4. HPLC analysis of (A) wild-type *R. jostii* RHA1 and (B) Δ*pcaHG* markerless deletion strains grown in M9 minimal media containing 0.1% 4-hydroxybenzoic acid (authentic standard, C) as sole carbon source, grown for 48 hr at 30 ^o^C, showing the accumulation of protocatechuic acid in the gene deletion strain, but not in the wild-type strain. Y-axis, mAU (270 nm); x-axis retention time (min).

Figure S5. Effect of *pcaHG* gene deletion on production of 2,4-PDCA (upper panels) and 2,5-PDCA (lower panels) on M9 media containing 0.1% 4-hydroxybenzoic acid and 0.4% glucose after 144 hr at 30 ^o^C, observed by HPLC analysis. Upper panels: wild-type *R. jostii* RHA1 containing pTipQC2ligAB (upper left); *R. jostii* Δ*pcaHG* containing pTipQC2ligAB (upper middle, 486 mg/L, also visible peaks for 4-hydroxybenzoic acid and protocatechuic acid); 2,4-PDCA standard. Lower panels: wild-type *R. jostii* RHA1 containing pTipQC2praA (lower left); *R. jostii* Δ*pcaHG* containing pTipQC2ligAB (upper middle, 810 mg/L); 2,5-PDCA standard. Y-axis, mAU (270 nm); x-axis retention time (min).


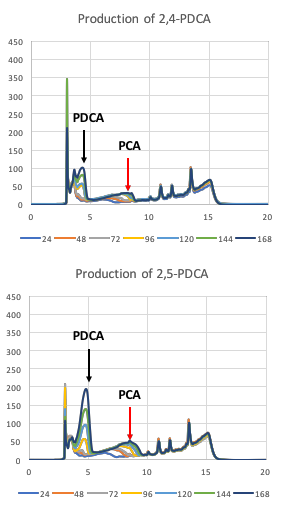


Figure S6. HPLC traces showing production of 2,4-PDCA by *R. jostii* Δ*pcaHG* containing pTipQC2ligAB (upper panel, 200 mg/L after 168 hr), and production of 2,5-PDCA by *R. jostii* Δ*pcaHG* containing pTipQC2praA (lower panel, 287 mg/L after 168 hr), from M9 minimal media containing 1% wheat straw lignocellulose, grown for 24-168 hr at 30 ^o^C. Y-axis, mAU (270 nm); x-axis retention time (min).


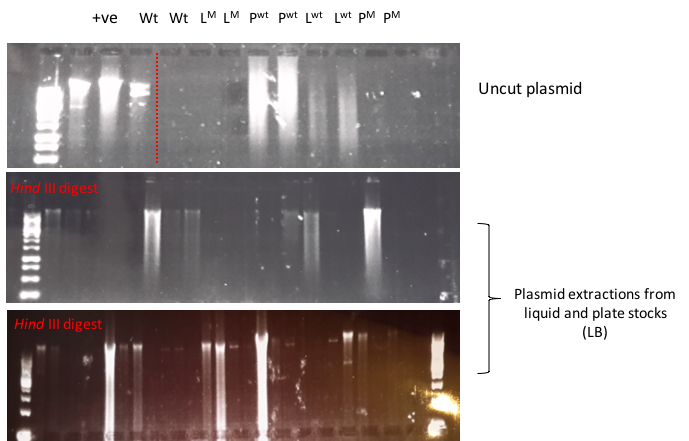


Figure S7. Agarose gels showing loss of plasmid DNA (lanes containing no visible bands) from *R. jostii* Δ*pcaHG*-pTipQC2ligAB, from liquid and plate stocks, over 2-3 weeks.


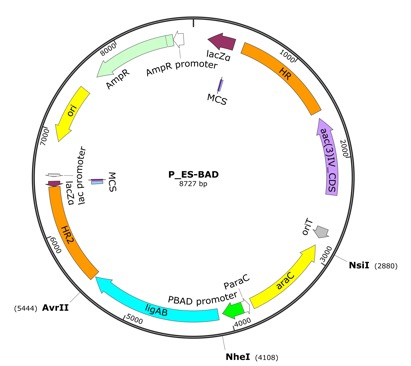


**A**

**B**


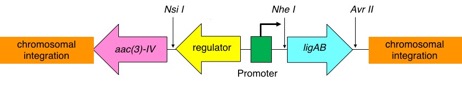


Figure S8. A. Chromosomal integration vector for insertion of *ligAB* or *praA* genes onto *R. jostii* RHA1 chromosome, with or without a promoter sequence. B. Construct for chromosomal expression of *ligAB* genes under the control of a gene promoter.

| **Primer name** | **Primer sequence 5’-3’** |
| --- | --- |
| Fragment 1.FOR | CTTGCATGCCTGCAGGTCGACTCTAGAGGATCCCCGAGGAGAAGAACGCGCCGCC |
| Fragment 1.REV | AACTTCGAAGCAGCTCCAGCCTACAATGGTCAACAAAGTCTTCGC |
| Fragment 2.FOR | CCGTTGCGAAGACTTTGTTGACCATTGTAGGCTGGAGCTGCTTCG |
| Fragment 2.REV | TGATGTAGCCGTCAAGTTGTCATAAATGCATATTCCGGGGATCCGTCGACC |
| Fragment 3.FOR | CTGCAGGTCGACGGATCCCCGGAATATGCATTTATGACAACTTGACGGCTA |
| Fragment 3.REV | CGATTCTCTCTTTCTTCTCGGTCATGCTAGCATTAATTAATAACCTCCTTAGAGCTCGAATTCC |
| Fragment 4.FOR | GGAATTCGAGCTCTAAGGAGGTTATTAATTAATGCTAGCATGACCGAGAAGAAAGAGAG |
| Fragment 4.REV | CGACACCTTTCTGCGTTGTGACCGACCTAGGTCAGGCCTGGGCCAGGCTGTG |
| Fragment 5.FOR | CGGACACAGCCTGGCCCAGGCCTGACCTAGGTCGGTCACAACGCAGAAAGG |
| Fragment 5.REV | TAAAACGACGGCCAGTGAATTCGAGCTCGGTACCCCGCGGCGCGCTGCTGCTCGT |
| ligAB_F3 | GGCGGGAATGTCTATTTCCT |
| ligAB_R1 | CCGAGGCATGGTCGTTATAG |

Figure S9. PCR primers for construction of chromosomal integration vector in Figure S8.

| **Clone** | **mutation** | **Media** | **Time** | **Gene expression** |
| --- | --- | --- | --- | --- |
| clone 2 | 04166:*praA* | M9/0.1%PCA | 192 hr | 0.0083 (*praA*) |
| clone 5 | *pcaHG:praA* | M9/0.1%PCA | 192 hr | 0.0025 (*praA*) |
| clone 20-1 | 04166:*ligAB* | M9/0.1%PCA | 192 hr | 0.0065 (*ligAB*) |
| wild type | none | M9/0.1%PCA | 192 hr | None for *ligAB*  0.1461 (*pcaH)* |
| clone 1 | pcaHG:*ligAB* | M9/0.1%VA | 144 hr | 0.00015 (*ligAB*) |

Figure S10. Gene expression data for *praA* and *ligAB* genes in chromosomal gene insertion constructs (without promoter) by quantitative RT-PCR, showing very low expression of *praA* & *ligAB*, compared with wild-type *pcaH*.

PtipA TGTACATATCGAGGCGGGCTCCCACGGCCG -100 CCCGGGCTGAGGGAGCCGACGGCACGCGGCGGCTCACGGCGTGGCACGCG -50 GAACGTCCGGGCTTGCACCTCACGTCACGTGAGGAGGCAGCGTGGACGGC start

Ptpc5 GTATAATGGACGGC

Figure S11. Nucleotide sequence of P_tpc5_ (designated P_nit_ in Nakashima & Tamura 2004), which is a constitutive promoter based upon the inducible P_tipA_ promoter, but containing 5 mutations in -10 box

Figure S12. Intracellular LigAB activity from *R. jostii* *pcaHG*:*ligAB*(P_icl_) construct, grown in LB media containing 1-8% methanol for 24 hr. Assays were carried out after cell lysis (protein concentration 0.32 mg/ml) as described in Methods section, absorbance measured at 410 nm after 10 min assay. Y-axis, absorbance change at 410 nm.


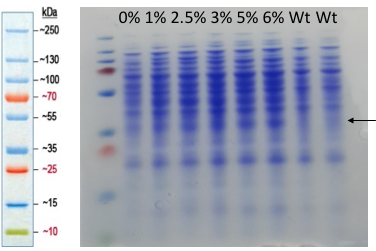


Figure S13. SDS-PAGE of cell lysates from *R. jostii* *pcaHG*:*ligAB*(P_icl_) construct, induced with 1-8% methanol, compared with wild-type *R. jostii* RHA1 (Wt), showing overexpression of LigB (33 kDa, marked with arrow).


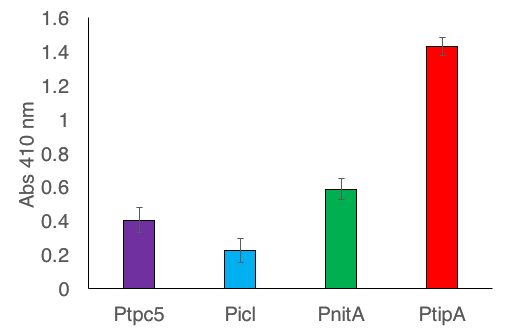


Figure S14. Intracellular LigAB activity observed using *R. jostii* *pcaHG*:*ligAB* constructs containing four different promoters for the *ligAB* genes, grown in LB media containing 5% methanol (P_icl_), 0.1% ε-caprolactam (P_nitA_), 0.1% thiostrepton (P_tipA_), or no additive (P_tpc5_) for 24 hr at 30 ^o^C. Assays were carried out after cell lysis as described in Methods section, absorbance measured at 410 nm after 10 min assay. Error bars indicate standard deviation from 3 biological replicates.

Figure S15. Whole cell biotransformation of protocatechuic acid (PCA) to 2,4-PDCA using *R. jostii* *pcaHG*:*ligAB(P_icl_)* and *R. jostii* *pcaHG*:*ligAB(P_tpc5_)* cells, compared with *R. jostii* Δ*pcaHG* as control, assessed by reverse phase HPLC. 0.5 mg/mL PCA (0.05%), 6 g/L NH_4_Cl, 160 mg *R. jostii* cells (grown on LB media) in 50 mM potassium phosphate buffer pH 7.5, total volume 4.0 mL. Incubated for 30 min at 30 ^o^C, aliquots taken at t = 0, 30 min, supernatant after centrifugation analysed by HPLC. Y-axis, mAU (270 nm); x-axis retention time (min).


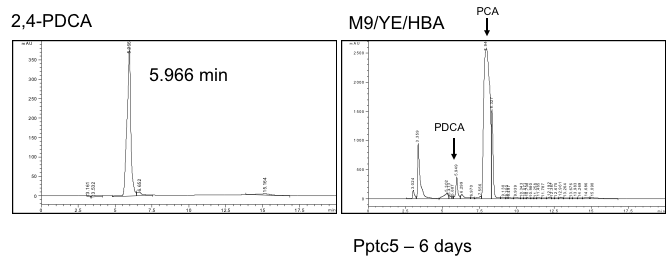


Figure S16. Production of 2,4-PDCA using *R. jostii* *pcaHG*:*ligAB(P_tpc5_)* grown on M9 minimal media containing 0.1% 4-hydroxybenzoic acid and 0.1% yeast extract for 144 hr at 30 ^o^C, supernatant after centrifugation analysed by HPLC. Left hand panel shows 2,4-PDCA authentic standard. Y-axis, mAU (270 nm); x-axis retention time (min).


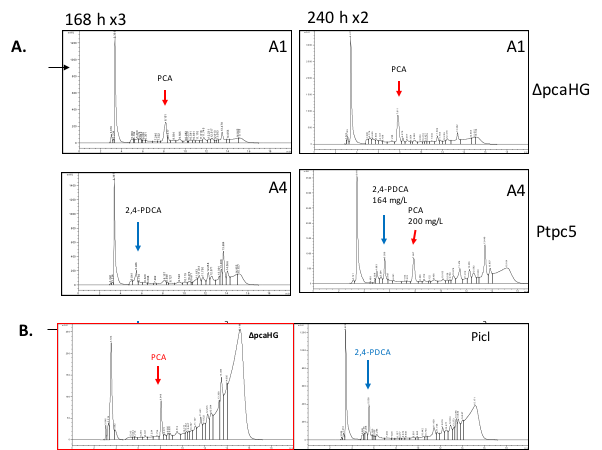


Figure S17. Production of 2,4-PDCA from M9 media containing 1% Green Value Protobind lignin by constructs containing chromosomal expression of *ligAB* genes, supernatant after centrifugation analysed by HPLC. A. Production of 2,4-PDCA and PCA by *R. jostii* *pcaHG*:*ligAB*(P_tpc5_), compared with *R. jostii* Δ*pcaHG* lacking the *ligAB* genes, grown in M9 media containing 1% Green Value Protobind lignin and 0.1% yeast extract after 168 and 240 hr at 30 ^o^C. B. Production by *R. jostii* *pcaHG*:*ligAB*(P_icl_), compared with *R. jostii* Δ*pcaHG* lacking the *ligAB* genes, grown in M9 media containing 1% Green Value Protobind lignin, 5% methanol and 0.1% yeast extract after 168 hr at 30 ^o^C. Y-axis, mAU (270 nm); x-axis retention time (min).

Figure S18. SDS-PAGE gel of cell extracts of *R. jostii* *pcaHG*:*ligAB*(P_tpc5_) with or without pTipQC2-dyp2, grown in LB broth for 24 hr at 30 ^o^C, induced with 0.1% thiostrepton, showing expression of recombinant Dyp2 peroxidase (marked with arrow).

Figure S19. Production of 2,4-PDCA by *R. jostii* *pcaHG*:*ligAB*(P_tpc5_) with or without pTipQC2-dyp2, grown on M9 minimal media containing 1% wheat straw lignocellulose for 120 hr at 30 ^o^C. 2,4-PDCA titres: 186 mg/L with pTipQC2-dyp2, 116 mg/L without pTipQC2-dyp2. Y-axis, mAU (270 nm).

Figure S20. Small scale (4 mL) testing of the production of 2,4-PDCA and protocatechuic acid (PCA) by *R. jostii* *pcaHG*:*ligAB*(P_tpc5_) with or without expression of *Amycolatopsis* *dyp2* gene, grown on M9 minimal media containing 0.1% or 0.2% Green Value Protobind lignin, testing the addition of 0.5-1.5 mM FeSO_4_, MnSO_4_, and CuSO_4_, and additional 0.5% NH_4_Cl. Incubated for 168 hr at 30 ^o^C, analysed by HPLC as described in Methods.


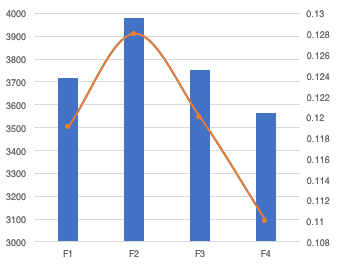


64 hr

43 hr

39 hr

20 hr

mAU

Figure S21. Production of 2,4-PDCA by HPLC (peak height in mAU) vs time from 2.5L bioreactor for *R. jostii* *pcaHG*:*ligAB*(P_tpc5_)-pTipQC2-dyp2, grown on M9 minimal media containing 1% Green Value Protobind lignin at 30 ^o^C for 20-64 hr, showing maximum titre at 39 hr.


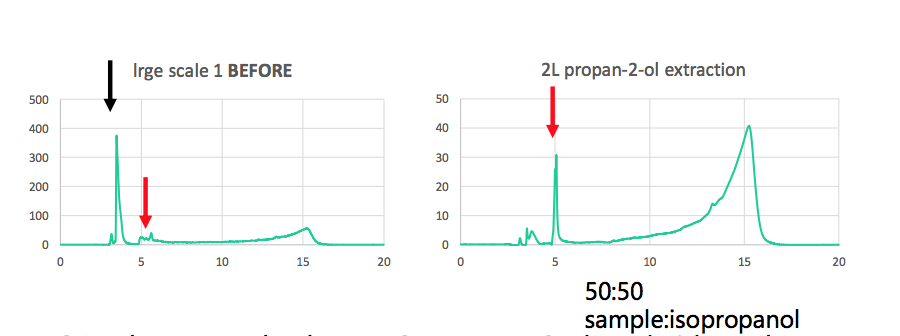


B

A

Figure S22. Extraction of 2,4-PDCA product (assessed by HPLC analysis, red arrow) from bioreactor fermentation broth (Panel A, *R. jostii* *pcaHG*:*ligAB*(P_tpc5_)-pTipQC2-dyp2 grown on M9 minimal media/1% Green Value Protobind lignin at 30 ^o^C for 40 hr) into isopropanol (Panel B, after isopropanol extraction), as described in Methods. Y-axis, mAU (270 nm); x-axis retention time (min).


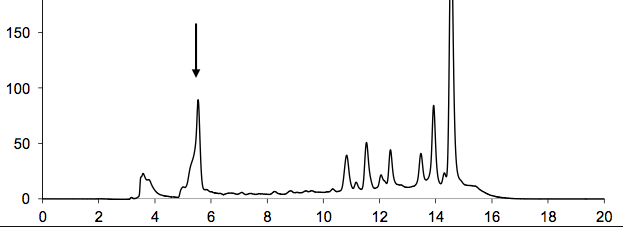


Figure S23. HPLC analysis of solid containing 2,4-PDCA product (arrow) isolated after solvent evaporation of isopropanol extract of *R. jostii* *pcaHG*:*ligAB*(P_tpc5_)-pTipQC2-dyp2 bioreactor fermentation. Y-axis, mAU (270 nm); x-axis retention time (min).


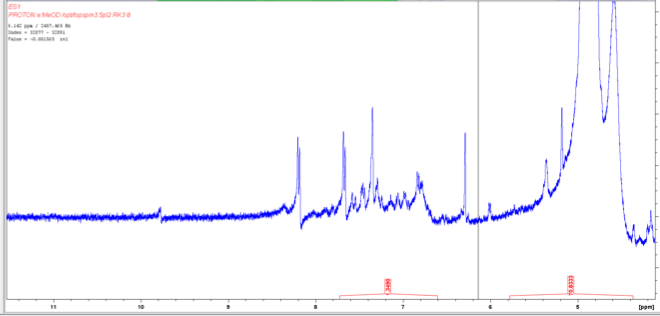


Figure S24. ^1^H NMR spectrum (300 MHz, d_4_-MeOH) of solid containing 2,4-PDCA isolated from *R. jostii* *pcaHG*:*ligAB*(P_tpc5_)-pTipQC2-dyp2 bioreactor fermentation after isopropanol extraction. Assignment of 2,4-PDCA ^1^H signals marked with arrows: δ_H_ 8.22 (1H, d, J = 7 Hz, H-6), 7.65 (1H, d, J = 7 Hz, H-5), 7.32 (1H, s, H-3) ppm.
